# mRNA prime-boost vaccination promotes clonal continuity in germinal center reactions and broadens SARS-CoV-2 variant coverage

**DOI:** 10.1016/j.ymthe.2025.08.008

**Published:** 2025-08-08

**Authors:** Matias Ciancaglini, Jonas Fixemer, Cemre Seven, Mirela Dimitrova, Davide Finozzi, Denice Weklak, Anna Lena Kastner, Franziska Jönsson, Ingrid Wagner, Ilena Vincenti, Anneli Peters, Maddy L. Newby, Max Crispin, Doron Merkler, Florian Kreppel, Daniel D. Pinschewer

**Affiliations:** 1 Department of Biomedicine, University of Basel, Petersplatz 10, 4051 Basel, Switzerland; 2 Institute for Biochemistry and Molecular Medicine, Center for Biomedical Education and Research (ZBAF), Department of Human Medicine, Faculty of Health, Witten/Herdecke University, 58453 Witten, Germany; 3 Department of Pathology and Immunology, University of Geneva, Geneva, Switzerland; 4 Institute of Clinical Neuroimmunology, University Hospital Ludwig-Maximilians-Universität München, Munich, Germany; 5 Biomedical Center (BMC), Faculty of Medicine, Ludwig-Maximilians-Universität München, Martinsried, Germany; 6 School of Biological Sciences, University of Southampton, SO17 1BJ Southampton, UK; 7 Division of Clinical Pathology, Geneva University Hospital, 1211 Geneva, Switzerland

**Keywords:** SARS-CoV-2, vaccination, germinal centers, neutralizing antibodies, mRNA vaccines, prime-boost immunization, clonal selection, variant coverage, adenovirus vector

## Abstract

mRNA- and adenovirus-based (rAd) SARS-CoV-2 vaccines have been widely used in various homologous and heterologous prime-boost combinations. It remains unknown, however, which vaccination regimens effectively promote B cell clonal continuity (i.e., the participation of individual B cell clones in durable GC reactions to prime and subsequent boost). Here, we characterize SARS-CoV-2-specific GC B cell dynamics in vaccinated mice. While rAd/mRNA prime-boost elicited SARS-CoV-2-neutralizing antibody (nAb) titers equivalent to those induced by mRNA/mRNA, the latter provided broader nAb coverage of variants of concern. B cell fate-mapping experiments revealed that mRNA/mRNA afforded higher clonal continuity between primary and secondary GC reactions than rAd/mRNA, which was associated with similarly sized but more durable GC responses upon mRNA prime. Homologous rAd/rAd prime-boost resulted in vector backbone-biased antibody responses and limited clonal continuity of SARS-CoV-2-specific B cells. Our study suggests that high clonal continuity as observed during mRNA homologous prime-boost is key for vaccination regimens against rapidly evolving pathogens.

## Graphical abstract

**Figure:**
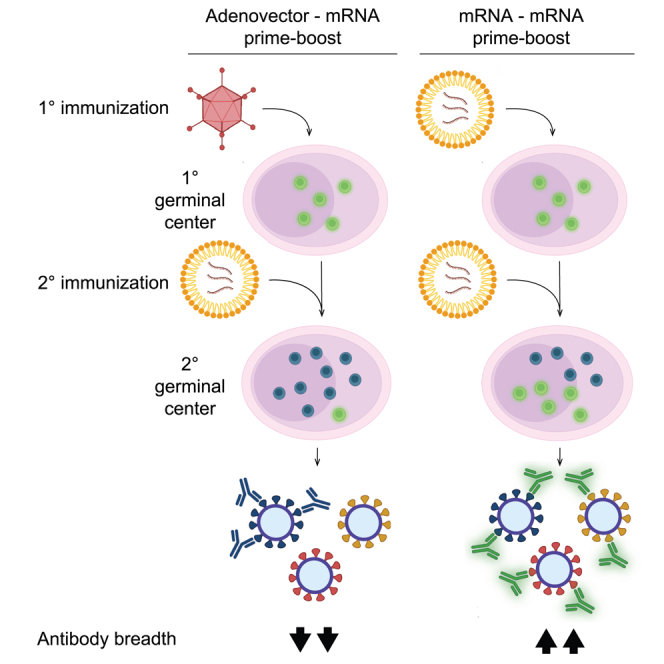

## Teaser

Ciancaglini and colleagues show that while homologous and heterologous combinations of mRNA- and adenovirus-based SARS-CoV-2 vaccines produce similar neutralizing antibody levels, homologous mRNA prime-boost elicits more durable germinal center reactions, a more continuous evolution of individual B clones throughout prime and boost, and that it offers better coverage of variants of concern.

## Introduction

Effective and long-lived antibody responses to vaccination generally depend on germinal center (GC) reactions.^1^ Affinity maturation yields long-lived plasma cells and memory B cells (MBCs), which can serve as a source of durable antibody immunity and mount rapid recall responses upon re-infection, respectively. Rapidly evolving pathogens such as SARS-CoV-2^2^ may evade control by preexisting antibodies, necessitating the formation of a variant-adapted response. While low- as well as high-affinity GC B cells are recruited into the pool of antibody-secreting cells (ASCs),^3^^,^^4^ MBCs are generally less hypermutated and of lower affinity than ASCs^5^^,^^6^; however, their diversity tends to cover a broader range of antigenic variants.^7^^,^^8^^,^^9^ Upon encounter with viral mutants, suitable MBC clones can rapidly proliferate and differentiate into ASCs, thus offering a timely defense. In the context of prime-boost vaccination, it is expected that the breadth of antibody and MBC immunity can be augmented when MBCs induced during primary immunization re-enter GCs upon booster vaccination. Thus, these cells undergo additional rounds of antigen-driven affinity maturation and diversification.^10^^,^^11^ Alternatively, B cells may persist inside long-lived GCs throughout prime and boost immunization, which is also expected to result in an extended evolutionary trajectory of the respective clones.^12^ Both mechanisms may thus jointly equip the host with highly diverse MBC clones to warrant a timely high-affinity antibody response upon a future encounter with new viral variants.^2^

Vaccination campaigns against the SARS-CoV-2 pandemic have relied on a diverse collection of vaccine technologies. mRNA- and adenoviral vector-based products such as mRNA-1273 and ChAdOx1 nCoV-19, respectively, were among the most effective and widely used.^13^ These vaccines express the spike protein of SARS-CoV-2 to elicit both T cells and virus-neutralizing antibodies, with the latter representing the main correlate of protection against symptomatic infection,^14^ whereas T cell immunity may prevent severe or life-threatening disease.^15^^,^^16^ Both vaccine platforms induce GC reactions that can be boosted upon secondary immunization.^17^^,^^18^ Owing to the risk of very rare but severe side effects associated with the ChAdOx1 vaccine^19^ and occasionally as a result of local supply chain bottlenecks, many recipients of a ChAdOx1 priming dose were eventually boosted with an mRNA vaccine.^20^ By consequence also, the reverse order of mRNA prime followed by ChAdOx1 boost was only rarely used^21^^,^^22^^,^^23^^,^^24^^,^^25^^,^^26^^,^^27^ Interestingly, the heterologous ChAdOx1/mRNA prime-boost regimen elicited equal or higher antibody titers than ChAdOx1- or mRNA-based homologous prime-boost vaccination regimens.^23^^,^^24^^,^^25^^,^^28^ Limited knowledge exists, however, regarding how the choice of vaccines that are combined for prime and boost impacts on the breadth of the antibody response elicited.^26^ Moreover, we sorely lack an understanding of how different vaccination regimens compare in terms of clonal continuity—in other words, the ability to engage individual B cell clones in durable GC responses to prime and subsequent boost^29^^,^^30^ and how this parameter may relate to the antigenic breadth of vaccination-induced antibody immunity.

In this study, we characterized and compared the induction of SARS-CoV-2 spike-specific GC B cell responses to mRNA and ChAdOx1 vaccines when administered to mice in homologous and heterologous prime-boost regimens. While eliciting comparable levels of total spike-specific antibody titers, the antibody response to mRNA/mRNA prime-boost vaccination was broader than the one to ChAdOx1/mRNA—it was better able to cross-neutralize variants of concern. Fate-mapping experiments revealed that mRNA/mRNA homologous prime-boost afforded higher clonal continuity between primary and secondary GCs than ChAdOx1/mRNA heterologous prime-boost.

## Results

### Spike-specific B cell responses to mRNA-1273 prime are longer lived than those to ChAdOx1

**Figure 1:**
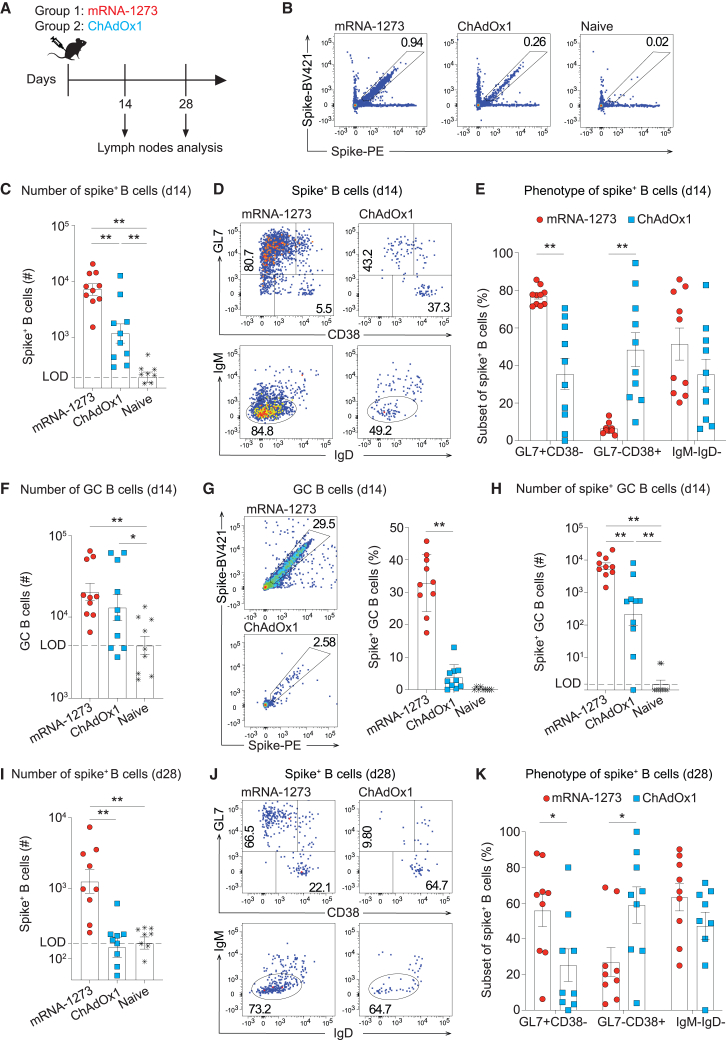
Spike-specific B cell responses to mRNA-1273 prime are longer lived than responses to ChAdOx1 (A) Experimental design. Mice received mRNA-1273 or ChAdOx-1 i.m. on day 0. Inguinal lymph nodes (iLNs) were collected on days 14 and 28. (B and C) Exemplary FACS plots and absolute numbers of spike^+^ B cells in iLNs on day 14 after mRNA-1273 or ChAdOx-1 vaccination. (D and E) Exemplary FACS plots and percentages of GC phenotype (GL7^+^CD38^−^), memory phenotype (GL7^−^CD38^+^), and class-switched (IgM^−^IgD^−^) cells among spike^+^ B cells on day 14. (F) Total numbers of GC B cells in iLNs on day 14. (G) Exemplary FACS plots and percentages of spike^+^ B cells within the GC compartment on day 14. (H) Total number of spike^+^ GC B cells in iLNs on day 14. (I) Spike^+^ B cells in iLNs on day 28. (J and K) Exemplary FACS plots and percentages of GC phenotype (GL7^+^CD38^−^), memory phenotype (GL7^−^CD38^+^) and class-switched (IgM^−^IgD^−^) cells among spike^+^ B cells on day 28. Symbols represent individual mice, with bars in (C, E–I and K) showing the mean ± standard error of the mean (SEM). Results from two independent experiments are displayed. One-way ANOVA with Tukey’s post-test was used for statistical analysis. ∗*p* < 0.05; ∗∗*p* < 0.01; *p* > 0.05 was considered not statistically significant and is not indicated.

We compared the spike-specific B cell response elicited upon intramuscular (i.m.) immunization of C57BL/6 mice with mRNA-1273 and ChAdOx1 (Figures 1A and S1). Spike protein tetramers were used to enumerate and characterize spike-binding (spike^+^) B cells in inguinal lymph nodes (iLNs), draining the site of immunization, on days 14 and 28 of the experiment (Figures 1A, 1B, and S2A). By day 14, both vaccines had triggered spike^+^ B cell numbers exceeding unimmunized (naive) backgrounds, yet mRNA-1273-induced responses were ∼10 times more numerous than those elicited upon ChAdOx1 vaccination (Figures 1B and 1C). The use of an receptor-binding domain (RBD) tetramer instead of the spike tetramer resulted in an approximately proportional reduction in the frequencies of specific B cells detected, irrespective of the vaccine platform used for immunization (Figures S2H and S2I). A variable but on average comparable proportion of spike^+^ B cells was class switched (immunoglobulins IgM^–^IgD^–^) in the two immunization regimens, whereas an analysis of GL7 and CD38 expression revealed clear differences (Figures 1D and 1E). On day 14 after mRNA-1273 prime, the spike^+^ B cell response consisted mostly of GL7^+^CD38^–^ GC B cells with proportionally fewer GL7^–^CD38^+^ MBCs, while the two subsets were approximately evenly represented after ChAdOx1 immunization. We noted that in ChAdOx1-vaccinated mice, the proportion of GC phenotype cells among spike^+^ B cells varied substantially, supposedly reflecting asynchronous GC responses in different animals. When enumerating total GC B cell numbers irrespective of B cell specificity, the responses elicited by the two vaccines were of comparable magnitude (Figure 1F). Accordingly, the proportion of spike^+^ cells among GC B cells was significantly higher upon mRNA-1273 immunization than upon ChAdOx1 vaccination (Figure 1G), and the total number of spike^+^ GC B cells in mRNA-1273-vaccinated mice exceeded the respective counts in ChAdOx1-vaccinated animals by >10-fold (Figure 1H). This discrepancy between comparable total GC B cell numbers but vastly differential spike^+^ GC B cell counts implied that a substantial proportion of the ChAdOx1-induced GC B cell compartment was directed against antigens other than spike, a response that likely involved adenoviral backbone antigens.^31^^,^^32^^,^^33^

By day 28 after immunization, spike^+^ B cell responses to both mRNA-1273 and ChAdOx1 had contracted ∼10-fold (Figure 1I; compare Figure 1C), and in recipients of the latter vaccine spike^+^ B cells were again down in the background range of unimmunized controls. The proportion of GC phenotype cells among spike^+^ B cells of mRNA-1273-vaccinated mice declined somewhat between days 14 and 28, but a population of spike^+^ GC B cells remained detectable for up to 42 days after prime (Figures 1J and 1K; compare Figures 1D, 1E, and S2D–S2G). Altogether these data showed that mRNA-1273 induced a spike-specific B cell response that reached higher peak numbers and was detectable for longer periods of time than ChAdOx1 vaccination. Moreover, the former response comprised a higher proportion of spike^+^ B cells.

### mRNA-1273/mRNA-1273 and ChAdOx1/mRNA-1273 prime-boost regimens induce GC B cell responses of similar magnitude

**Figure 2:**
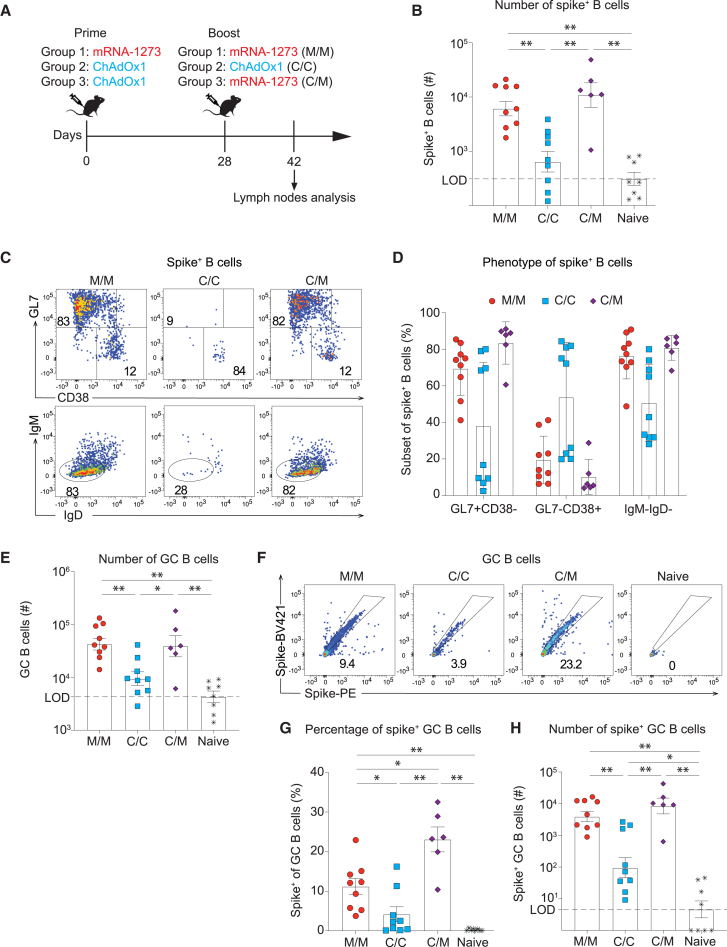
mRNA-1273/mRNA-1273 and ChAdOx1/mRNA-1273 prime-boost regimens induce GC B cell responses of similar magnitude (A) Experimental design. Mice were primed and boosted on days 0 and 28 with the indicated combinations of mRNA-1273 and ChAdOx-1. iLNs were collected on day 42 for analysis by flow cytometry. (B) Total numbers of spike^+^ B cells in iLNs on day 42. (C and D) Exemplary FACS plots and percentages of GC phenotype (GL7^+^CD38^−^), memory phenotype (GL7^−^CD38^+^), and class-switched (IgM^−^IgD^−^) spike^+^ B cells. (E) Total numbers of GC B cells in iLNs. (F–H) Exemplary FACS plots, percentages, and absolute numbers of spike^+^ GC B cells. (G) For statistical analysis, one-way ANOVA with Tukey’s post-test was performed. Symbols represent individual mice, with bars in (B, D, E, G, and H) showing the mean ± SEM. ∗*p* < 0.05; ∗∗*p* < 0.01; *p* > 0.05 was considered not statistically significant and is not indicated.

Next, we compared the immunogenicity of mRNA-1273/mRNA-1273 homologous (M/M), ChAdOx1/ChAdOx1 homologous (C/C), and ChAdOx1/mRNA-1273 heterologous (C/M) prime-boost vaccination, three regimens that have been used frequently during the COVID-19 pandemic (see above).^21^^,^^22^^,^^23^^,^^24^^,^^25^^,^^26^^,^^27^ Prime and boost were administered i.m. at a 4-week interval (Figure 2A). Two weeks after secondary immunization, the two mRNA-boosted groups harbored comparable total numbers of GC B cells in the draining LN, while the response upon homologous ChAdOx1 boost was significantly lower (Figure 2B). Most of the spike-specific B cells in the two mRNA-boosted groups exhibited a GC phenotype, with a comparably minor subset of spike^+^ MBCs, and most cells were class switched (Figures 2C and 2D). In C/C-immunized animals the proportion of spike^+^ B cells that had undergone class switch and the cells’ repartition between GC and MBC phenotypes varied considerably as observed in two independent experiments. The total count of GC B cells and spike^+^ GC B cells was comparable in the two mRNA-boosted groups, significantly exceeding the respective numbers in homologous ChAdOx1 primed and boosted mice (Figures 2E–2H). Interestingly, the proportion of spike^+^ cells among GC B cells also was significantly higher when mRNA-1273 was used for boost (Figures 2F and 2G), suggesting that the vast majority of the GC response elicited by ChAdOx1 homologous prime-boost was directed to antigens other than spike.

Spike-specific CD8 and CD4 T cell responses were analyzed from spleen by major histocompatibility complex (MHC) class I tetramer staining and by activation-induced marker (AIM) assays, respectively (Figure S3). Tetramer-binding CD8 T cell responses were readily detected after primary immunization and were significantly higher in response to ChAdOx1 than upon mRNA-1273 vaccination (Figures S3A and S3B). In contrast, spike-specific CD4 T cell responses to prime immunization were close to detection limits, irrespective of the vaccine administered (Figures S3C–S3E). mRNA-1273 homologous prime-boost and C/M heterologous prime-boost induced similar numbers of spike-specific CD8 T cells. Both of these responses were significantly higher than those elicited by ChAdOx1 homologous prime-boost (Figure S3B). When analyzed after the boost, an analogous hierarchy in the magnitude of responses was observed for spike-specific CD4 T cells and for the CXCR5^+^PD-1^+^ T follicular helper subset contained therein (Figures S3D and S3E). Altogether these data documented that M/M and C/M prime-boost induced higher numbers of spike-specific CD4 and CD8 T cell responses than ChAdOx1 homologous prime-boost.

### Homologous mRNA-1273 prime-boost vaccination affords superior clonal continuity in GC B cell responses

**Figure 3:**
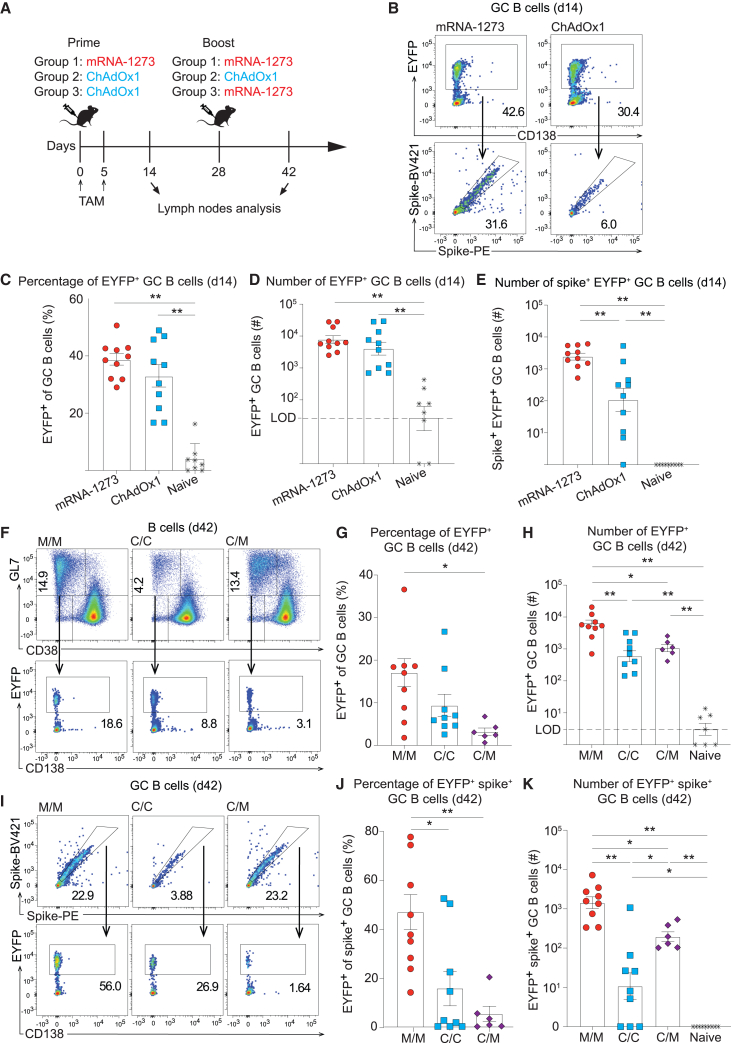
Homologous mRNA-1273 prime-boost vaccination affords superior clonal continuity in GC B cell responses (A) Experimental design. AID^rep^ mice were immunized on day 0 and treated with tamoxifen on days 0 and 5. Secondary immunization was performed on day 28. iLNs were analyzed on days 14 and 42. (B) Exemplary FACS plots of fate-mapped GC B cells (EYFP^+^GL7^+^CD38^−^, top row) and spike^+^ B cells within this population (bottom row) on day 14. (C and D) Percentages and absolute numbers of fate-mapped cells among GC B cells on day 14. (E) Total number of spike^+^ fate-mapped GC B cells on day 14. (F) Exemplary FACS plots of GC B cells (GL7^+^CD38^−^, top row) and fate-mapped B cells within this population (bottom row) on day 42. (G and H) Percentages and total number of fate-mapped cells among GC B cells on day 42. (I) Representative FACS plots of spike^+^ GC B cells (top row) and fate-mapped B cells within this population (bottom row) at day 42. (J and K) Percentages and absolute number of fate-mapped cells among spike^+^ GC B cells on day 42. One-way ANOVA with Tukey’s post-test was performed for statistical analysis.Symbols represent individual mice, with bars in (C, D, E, G, H, J, and K) showing the mean ± SEM. ∗*p* < 0.05; ∗∗*p* < 0.01; *p* > 0.05 was considered not statistically significant and is not indicated.

To compare the clonal continuity of different prime-boost regimens—in other words, their ability to engage individual B cell clones in a long-term GC response to prime and subsequent boost^29^^,^^30^—we exploited activation-induced cytidine deaminase (AID) fate-mapping reporter mice (AID^rep^).^12^ Administration of tamoxifen to AID^rep^ mice results in the irreversible enhanced yellow fluorescent protein (EYFP) labeling of B cells expressing AID during a defined time window of the immune response. This EYFP label is transmitted to all labeled cells’ progeny, allowing their tracing in the context of subsequent responses. We primed AID^rep^ mice with either mRNA-1273 or ChAdOx1 and administered tamoxifen to label activated B cells during the first 5 days of the response (Figure 3A). On day 14 after primary vaccination ∼20%–50% of GC B cells expressed EYFP irrespective of the vaccine administered, and also total numbers of EYFP-expressing GC B cells were in comparable ranges (Figures 3B–3D and S2B). Notably, however, and in line with the results reported in Figure 1, the proportion of spike-specific B cells among EYFP^+^ GC B cells and the total number of EYFP^+^ spike^+^ GC B cells were higher in animals undergoing mRNA prime than in those receiving the ChAdOx1 vaccine (Figures 3B–3E). Next, we used the AID^rep^ fate-mapping approach to readout clonal continuity between GC responses to prime and boost. The proportion of EYFP^+^ secondary GC B cells (i.e., the proportion of cells in the GC response to boost that had been activated and labeled during prime vaccination) was highest upon M/M prime-boost and lowest after C/M heterologous prime-boost, with C/C-vaccinated animals somewhere in between (Figures 3F, 3G, and S2C). Accordingly, absolute numbers of EYFP^+^ secondary GC B cells in M/M prime-boost were significantly higher than in either of the other two groups (Figure 3H). Moreover, EYFP^+^ secondary GC B cells made up for half of the spike^+^ GC B cell compartment in M/M-immunized animals, whereas only about ∼20% and ∼5% of these cells were obtained upon C/C or C/M vaccination, respectively (Figures 3I and 3J, bottom row). By consequence, M/M-vaccinated animals harbored ∼9.6- and ∼194.2-fold higher numbers of EYFP^+^ spike^+^ secondary GC B cells than mice undergoing either C/M or C/C prime-boost, respectively (Figure 3K). These results indicated that homologous M/M prime-boost immunization afforded substantial clonal continuity between spike-specific GC B cell responses to prime and boost, whereas booster responses upon heterologous C/M and homologous C/C vaccination were dominated by newly recruited clones. Prime vaccination into one leg and boosting on either the same side (ipsilateral) or on the opposite side (contralateral) can help to differentiate between GC responses formed by recirculating MBCs (contralateral as well as ipsilateral side) and antigen re-fueling of GCs upon boost (ipsilateral side only).^29^ We primed mice with mRNA-1273 followed by either ipsilateral or contralateral mRNA-1273 booster vaccination on day 28, and on day 42 we analyzed the LN draining the site of the booster vaccination (Figure S4A). The spike-specific GC B cell response to ipsilateral mRNA boost was on average ∼10-fold higher than the one in response to contralateral boost (Figure S4B). While not excluding the recruitment of MBCs back into GCs, these results suggested that most of the GC reaction upon ipsilateral M/M prime-boost resulted from antigen re-fueling of persisting GCs.

**Figure 4:**
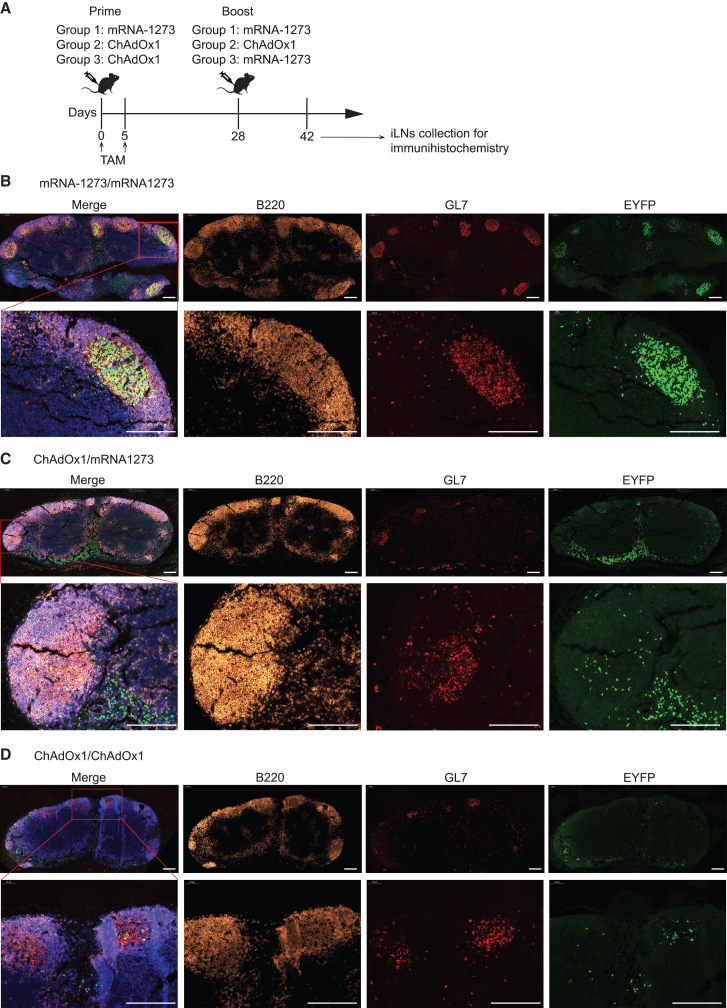
Homologous mRNA-1273 prime-boost vaccination affords superior clonal continuity in GC B cell responses (A) Experimental design. AID^rep^ mice were immunized on day 0 and treated with tamoxifen on days 0 and 5. Secondary immunization was performed on day 28 and iLNs were processed for immunohistochemistry on day 42. (B–D) iLNs sections from mice were primed and boosted with mRNA-1273/mRNA-1273, ChAdOx-1/mRNA-1273, or ChAdOx-1/ChAdOx-1. Sections were stained for B220, GL7, and EYFP (DAPI was included for detection of nuclei and is shown in the merge only). One representative section from a total of six mice per group, collected in two separate experiments, is shown. Scale bars: 200 μm.

To complement our flow cytometric assessment, we performed histological analyses on draining LNs of AID^rep^ mice that had been vaccinated with either M/M, C/C, or C/M and treated with tamoxifen as described above (Figure 4A). The labeling of GL7^+^ GC B cells and EYFP^+^ fate-mapped cells revealed that mRNA/mRNA prime-boost vaccination produced GCs that were densely populated by EYFP^+^ secondary GC B cells (Figure 4B; B220 is shown to demarcate the cortical B zone in LNs). In remarkable contrast, the GCs in draining LNs of mice undergoing ChAdOx1/mRNA or C/C immunization exhibited a relative paucity in EYFP^+^ B cells (Figures 4C and 4D). EYFP^+^ cells were also found in the medullary region of draining LNs, likely representing MBCs that had been activated during prime immunization and upon boost had differentiated into ASCs. These cells were particularly abundant in animals undergoing ChAdOx1/mRNA prime-boost, suggesting ChAdOx1-primed B cells were efficiently driven into ASC differentiation when boosted by mRNA. Taken together, our histological analyses corroborated and extended the conclusion that the ability of mRNA/mRNA prime-boost vaccination to promote clonal continuity of GCs between prime and boost was superior to ChAdOx1/mRNA or C/C vaccination.

### Antibody responses to homologous mRNA-1273 prime-boost exhibit broader variant coverage than those to heterologous C/M

**Figure 5:**
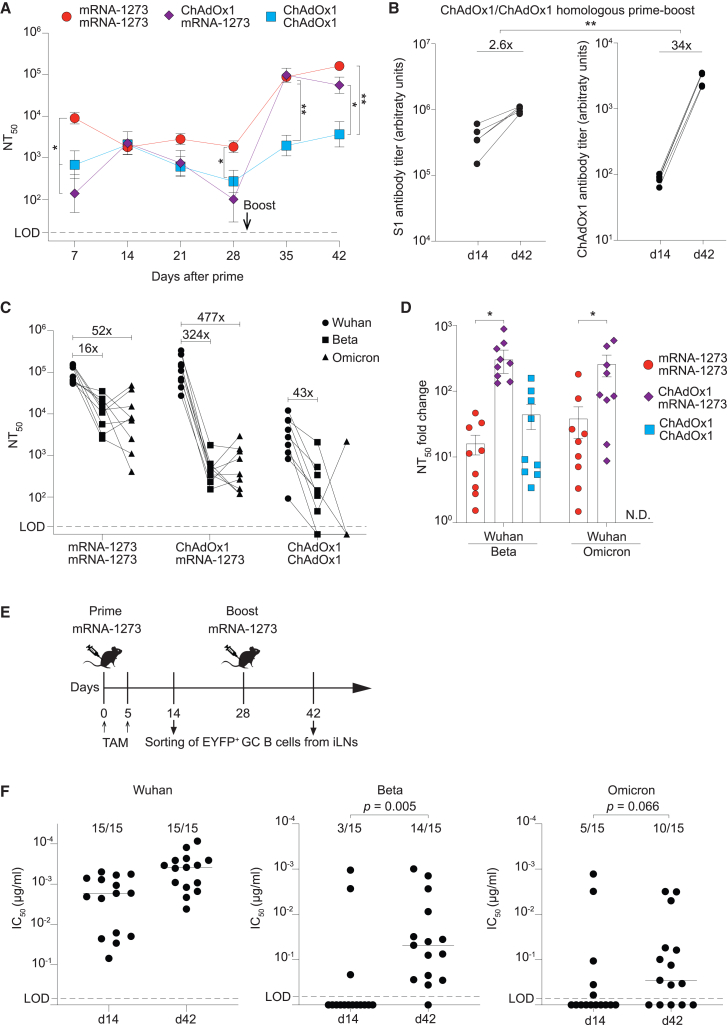
Antibody responses to homologous mRNA-1273 prime-boost exhibit broader variant coverage than those to heterologous ChAdOx1/mRNA-1273 Mice were immunized with combinations of mRNA-1273 and ChAdOx-1 on days 0 and 28 as in the experiments in Figures 2, 3, and 4. (A) Wuhan-Hu-1-specific SARS-CoV-2 neutralizing antibody titers over time. (B) SARS-CoV-2 spike S1 subunit-binding and ChAdOx-1 particle-binding antibody titers by ELISA on days 14 and 42. (C) Neutralizing activity in day 35 serum against Wuhan-Hu-1, Beta, and Omicron BA.5 variants of SARS-CoV-2 spike protein. (D) Conversion of the values displayed in (C) into a ratio of neutralizing serum titer (NT_50_) against Wuhan-Hu-1 SARS-CoV-2 spike over the NT_50_ against the Beta variant and into a ratio of (NT_50_) against Wuhan-Hu-1 SARS-CoV-2 spike over the NT_50_ against the Omicron BA.5 variants. (E) AID^rep^ mice were immunized with mRNA-1273 on day 0 and treated with tamoxifen on days 0 and 5. mRNA-1273 boost was performed on day 28. iLNs were collected from two mice each on days 14 and 42, and EYFP^+^ GC B cells were sorted onto feeder cells for single B cell cultures. (F) The analysis included clones that had been pre-screened for SARS-CoV-2 Wuhan-Hu-1-neutralizing activity, and a minimal antibody concentration. Symbols show neutralizing activity from individual single-cell culture supernatants (15 for each time point) against SARS-CoV-2 Wuhan-Hu-1 as well as against the Beta and Omicron variants. Two-way ANOVA with Tukey’s post-test was performed for statistical analysis in (A). Two-tailed paired Student’s t test was performed for statistical analysis in (B). Two-tailed unpaired Student’s t tests were performed for statistical analysis in (D). Data in (F) were analyzed by Fisher’s exact test, and *p* values are reported.Symbols in A represent the mean of each group, symbols in (B, C, D and F) represent individual mice, with bars in (D) showing the mean ± SEM.In (A), (B), and (D), ∗*p* < 0.05;∗∗*p* < 0.01; *p* > 0.05 was considered not statistically significant and is not indicated.

Next, we compared the kinetics of neutralizing antibody (nAb) induction in mice immunized with M/M, C/M, or C/C. At 1 week after immunization, mRNA-1273 prime yielded higher nAb titers than ChAdOx1 prime (Figure 5A). These differences evened out at week 2, but nAb titers in ChAdOx1-vaccinated animals exhibited a more pronounced decay up to the time point of boost at week 4 (Figure 5A). Irrespective of these differences, mRNA-1273 homologous or heterologous boost resulted in comparable nAb titers at week 5, which was in line with comparable numbers of spike-specific B cells in these two groups (Figure 5A; compare with Figure 2H). Homologous ChAdOx1 boost resulted in a comparably modest increase and lower resulting nAb titer than the other two vaccination regimens, as expected based on lower spike-specific and total GC B cell numbers. Anti-vector immunity has the potential to diminish immune responses to adenovirus vector-based vaccination,^31^^,^^32^^,^^34^ prompting us to perform ELISA assays for a comparison of total adenovector backbone- and spike-specific antibody responses. We found that ChAdOx1 homologous boost augmented vector backbone-binding antibodies 34-fold, whereas SARS-CoV-2 spike-specific antibody titers were augmented 2.6-fold only (Figure 5B). These findings raised the possibility that immunodominant adenoviral backbone-directed B cell responses competed with SARS-CoV-2-specific ones when ChAdOx1 was administered as homologous booster vaccination.

Besides nAb titers as a correlate of protection, the antigenic breadth and the coverage of SARS-CoV-2 variants is also of importance.^2^^,^^35^^,^^36^ Somatically hypermutated spike-specific B cells exhibit higher neutralizing breadth than those from their predecessors, suggesting the GC response augments the antigenic breadth of B cells over time.^37^^,^^38^ We compared the neutralizing activity of immune mouse sera elicited by the different Wuhan-Hu-1-based vaccination regimens against the Beta and Omicron BA.5 variants of SARS-CoV-2, which are known to evade a majority of Wuhan-Hu-1 spike-induced monoclonal nAbs.^35^^,^^36^ As expected, the serum of vaccinated mice exhibited more potent neutralizing activity against Wuhan-Hu-1 spike protein than against its Beta or Omicron variants (Figures 5C and 5D). Still, the relative reduction in neutralizing potency (i.e., the breadth of the response) varied greatly between different vaccination regimens. M/M-induced sera were on average 16-fold less potent against the Beta variant than against Wuhan-Hu-1, whereas in the C/M group, the respective difference was 324-fold. Analogously, the neutralizing potency of M/M-induced sera against Omicron was 52-fold lower than against Wuhan-Hu-1, while the respective difference for C/M-immune sera was 477-fold. As already indicated above, the sera of mice undergoing C/C homologous prime-boost vaccination exhibited substantially lower Wuhan-Hu-1-specific titers than those of M/M- or C/M-immunized mice. Accordingly, Beta variant-nAb titers were close to detection limits in many of our C/C-immunized animals, and neutralizing activity against Omicron was inconsistently detected, rendering the quantitative assessment of neutralizing breadth in this group less reliable.

To investigate whether the clonal continuity between primary and secondary GC reactions increased the breadth of the M/M-induced GC B cell repertoire, we primed and boosted AID^rep^ mice with mRNA-1273 and administered tamoxifen to label activated B cells during the first 5 days after prime vaccination. On day 14 after prime and on day 42 (14 days after boost), we sorted individual EYFP-labeled GC B cells onto a feeder cell layer promoting their clonal expansion and plasma cell differentiation (Figure 5E). From each time point of sampling we recovered 15 clones that produced Wuhan-Hu-1-neutralizing antibody at a high concentration. Only 4 out of 15 GC B cell clones sampled 2 weeks after prime neutralized the Beta variant to a detectable extent, whereas 14 out of 15 clones sampled 2 weeks after boost showed such activity (27% vs. 93%; *p* = 0.0005 by Fisher’s exact test [Figure 5F]). Following the same trend, only 5 out of 15 day 14 clones but 11 out of 15 day 42 clones exhibited Omicron-neutralizing activity (33% vs. 73%; *p* = 0.066). Taken together, our serological analyses revealed that M/M homologous prime-boost immunization elicited serum antibody responses of higher antigenic breadth than those induced by heterologous ChAdOx1/mRNA prime-boost. Moreover, our clonal analyses indicated that B cells participating in the response to mRNA-1273 prime and subsequently also in the GC reaction to homologous boost were enriched for variant-cross-reactive neutralizing activity.

## Discussion

This study reveals that the choice of how to combine mRNA- and adenovirus vector-based SARS-CoV-2 vaccines in prime and boost exerts a profound influence on the dynamics of spike-specific GC B cell responses. Moreover, it can profoundly influence the antigenic breadth of the resulting spike-nAb response, irrespective of total nAb titers reached. We confirm that both vaccines tested, mRNA-1273 and ChAdOx1, initiate robust GC reactions as a prerequisite for durable high-affinity antibody immunity against SARS-CoV-2.^39^^,^^40^ These GC reactions differ, however, in terms of durability, and our fate-mapping experiments document that the extent to which previously primed spike-specific B cells are included in the GC response after boost differs profoundly between different vaccination regimens. The observation that ipsilateral mRNA/mRNA prime-boost afforded stronger secondary GC reactions than contralateral boost supports the hypothesis that ipsilateral mRNA boost served primarily to re-fuel durable GCs,^29^^,^^41^ while the recruitment of MBCs back into newly formed GCs^10^^,^^12^^,^^42^ may play a proportionally minor role in this context. Accordingly, the more rapidly contracting GC response to ChAdOx1 may be less amenable to re-fueling, which could explain why the clonal continuity between primary and secondary GC responses in M/M vaccination was higher than in the C/M regimen.

How precisely these differences in clonal continuity between the two vaccination regimens are related to the differential antigenic breadth of serum antibody responses after boost deserves further investigation. The participation of individual B cell clones in more or less durable GC reactions after prime is expected to mostly define the repertoire of MBCs that can be recalled as immediate ASC output in the early phase after boost. It remains to be investigated, however, to what extent the re-fueled GC response may also contribute to the serum antibody response over time.^43^ The clonal analysis conducted in this study shows that B cell clones that have participated in primary and secondary GC reactions are on average more broadly reactive than those found in the primary GC. Vaccination regimens warranting substantial clonal continuity in B cell responses across prime and boost seem, therefore, optimally poised to promote antigenic breadth, which is important for vaccination against rapidly evolving pathogens. In keeping with these findings in mice, human GC reactions after mRNA prime-boost can last for months, engendering high levels of somatic hypermutation,^44^^,^^45^^,^^46^ and Wuhan-Hu-1 spike-based mRNA prime-boost of human vaccinees was found to elicit robust B cell and antibody responses against antigenic variants as distant as Omicron.^47^

Our study also provides mechanistic insights into the limitations of homologous ChAdOx1 prime-boost vaccination for antibody induction.^20^^,^^26^^,^^27^^,^^48^^,^^49^ As compared to mRNA-1273 prime, a lower proportion of ChAdOx1-induced EYFP^+^ fate-mapped GC B cells bound to spike, suggesting that a proportionally higher percentage of the GC reaction was dedicated to antigens other than spike. These responses likely comprise components of the adenoviral backbone. In support of this hypothesis, we detected vector-binding antibodies at 2 weeks after prime that were further augmented upon ChAdOx1 re-administration. Of interest, this increase in anti-vector antibodies upon homologous boost vastly exceeded the concomitant increase in spike^+^ antibodies. The reason for this differential boosting remains unexplored, but it likely relates to the mechanism of anti-vector immunity.^31^^,^^32^^,^^50^^,^^51^ Anti-adenovector antibodies can limit the level and duration of vectored transgene expression in a process that involves antibody effector mechanisms such as the intracellular Fc receptor TRIM21.^32^^,^^52^ While the SARS-CoV-2 spike protein is expressed only upon transduction of target cells in the vaccinee, a process that is subject to anti-vector antibody interference, significant amounts of adenoviral backbone proteins are formulated in the vaccine itself (compare to Figure S1A) and thus can boost the corresponding responses even if transduction of target cells is prevented or aborted. It seems likely, therefore, that lower immunogenicity of C/C as compared to ChAdOx1/mRNA is due, at least in part, to vector-specific antibodies interfering with target cell transduction upon secondary ChAdOx1 immunizations. The observation that even serologically distinct pairs of adenoviral vectors may not effectively boost upon each other suggests, however, that also vector backbone-specific T cell immunity can interfere with vector re-administration.^53^^,^^54^

In summary, our findings highlight that prime-boost vaccination for the induction of broad nAb immunity should induce long-lived GC reactions promoting the continuous evolution of specific B cell clones. While both mRNA and adenoviral vector-based vaccines effectively initiate GC reactions, differences in their longevity and clonal continuity upon boosting are notable. These parameters should be taken into consideration when selecting candidates for antibody-based vaccines against highly variable pathogens of global importance.

## Materials and methods

### Animal experiments, immunizations, and tamoxifen administration

C57BL/6 wild-type mice were originally purchased from Charles River Laboratories and were bred and kept under specific pathogen-free conditions for colony maintenance and experiments. AID^rep^ mice have been described^12^ and carry both a tamoxifen-inducible Cre recombinase (Cre-ERT2) gene in the *Aicda* locus and a Cre-inducible EYFP reporter gene in the ROSA26 locus. Experimental groups were sex and age matched. Mice were bred at the ETH Phenomics Center Zurich, and experiments were performed at the University of Basel in accordance with the Swiss law for animal protection and with the permission of the Cantonal Veterinary Office of Basel City.

Leftovers from clinically used mRNA-1273 (Moderna) vaccine aliquots were kindly provided by the Medical Polyclinic of Basel University Hospital, and aliquots were kept at −80°C until use.^55^ Immunizations were performed at a dose of 5 μg per mouse and administered i.m. in a volume of 40 μL into both rear hind limbs. ChAdOx1 was administered i.m. at a dose of 5 × 10^8^ viral particles (vp)/mouse in a volume of 40 μL into both rear hind limbs. These respective doses for mRNA-1273 and ChAdOx1 were chosen based on published evidence of the dose range within which these vaccine platforms elicit consistent high-level immune responses in mice.^18^^,^^56^^,^^57^^,^^58^^,^^59^

Tamoxifen was dissolved in corn oil at 20 mg/mL by shaking overnight at 37°C, protected from light. AID^rep^ mice were administered 100 μL of the tamoxifen corn oil mix intraperitoneally to induce Cre-mediated recombination.

### Production, purification, and characterization of ChAdOx1 vector preparations

ChAdOx1 viral vectors were propagated in standard HEK293 cells. Viral particles were purified from crude cell lysates (obtained by repeated freeze/thaw) by cesium chloride (CsCl) density gradient centrifugation. Two rounds of purification were carried out using discontinuous CsCl gradients with densities of 1.27 and 1.41 g/cm^3^. Vector-containing bands were isolated following centrifugation at 32,000 rpm for 2 h at 4°C. Purified preparations were desalted into an HEPES-based buffer (10 mM HEPES, 150 mM NaCl, pH 8.0) using PD-10 desalting columns (Cytiva, catalog no. 17085101) and supplemented with 10% (v/v) glycerol as a cryoprotectant. Vector titers were determined via SDS-mediated capsid disruption followed by UV absorbance measurement at 260 nm, as described by Mittereder et al.^60^ As a quality control for purity, samples were analyzed by SDS-PAGE, followed by silver staining according to Blum et al.^61^ To assess particle homogeneity, dynamic light scattering (DLS) measurements were performed. A total of 5 × 10^10^ vp were diluted in Ad-buffer (50 mM HEPES, 150 mM NaCl, pH 8.0) containing 1% (v/v) glycerol. Samples were measured in a square glass cuvette (PCS1115, Malvern Panalytical) using a Zetasizer Ultra Red (Malvern Panalytical). Three repeated multi-angle DLS (MADLS) measurements were performed under the following conditions: 25°C, 2 min equilibration time, and a fixed dispersant scattering mean count rate of 112 kcps. All remaining instrument parameters were set to automatic mode. “Z-average” and “polydispersity index” values were derived from 90° angle sub-measurements, while “peak size” was reported as obtained from MADLS analysis. Although representing a lab-scale production and not a clinical product, these data collectively demonstrated a high purity and homogeneity of the ChAdOx1 vector preparation consistent with Good Laboratory Practice-like quality standards and non-inferior in purity to vaccine batches in human clinical use.^62^

### Flow cytometry

Spleens and iLNs were mechanically disrupted and lymphocytes in single-cell suspensions were counted using an Immunospot S6 device (C.T.L.). For surface staining of B cells, iLN-derived cells were incubated with phycoerythrin (PE)- and BV421-conjugated spike tetramers for 1 h at 4°C. Samples were washed and subsequently stained for surface markers using the following antibodies: CD45R/B220 (RA3-6B2), CD138 (281-2), anti-MU/HU GL7 Antigen (GL7), CD38 (REA616), IgM (II/41), and IgD (11-26c.2a). For detection of S1-specific CD8 T cells, PE-conjugated H2-K^b^ tetramers were loaded with the SARS-CoV-2 spike epitope (VNFNFNGL). Peptide-MHC tetramers were prepared by the University of Lausanne tetramer core facility and were added to the antibody mix for staining. The antibody mix included the following antibodies: CD45R/B220 (RA3-6B2), CD8 (53-6.7), CD44 (IM7), CD62L (MEL-14), CD127 (A7R34), Klrg1 (2F1), CX3CR1 (SA011F11), CD27 (LG3A10), and CD43 (1B11) purchased from BioLegend. For the detection of spike-specific CD4 T cells by means of the activation-induced marker (AIM) assay, splenocytes were resuspended in RPMI media containing rat IgG (Sigma, catalog no. I4131), anti-mouse CD16/CD32 (2.4G2; Bio X Cell, catalog no. BE0307), and anti-CD154 (CD40L) antibody (BioLegend, clone: MR1) and incubated for 6 h at 37°C with no peptide stimulation or stimulation and S1 and S2 peptide pools purchased from GenScript (catalog no. RP30020). Peptide pools were used at a final concentration of 2 mg/mL for each peptide. Subsequent to stimulation, the cells were washed with FACS buffer (PBS supplemented with 2% fetal calf serum [FCS]) and resuspended in BD FcBlock (clone 2.4G2) for 5 min at room temperature (RT) prior to staining with a surface stain antibody cocktail containing the following specificities purchased from BioLegend: CD3 (145-2C11), CD4 (RM4-5), I-A/I-E (M5/114.15.2) PE, CD44 (IM7), CD62L (MEL-14), CXCR5 (L138D7), PD-1 (29F.1A12), and CD69 (H1.2F3). Dead cells were stained with Zombie-UV Fixable Viability Kit (BioLegend, catalog no. 423107). All samples were fixed by incubation with 2% paraformaldehyde (PFA) for 15 min at RT and measured on a LSRFortessa flow cytometer (Becton Dickinson). Data were analyzed with FlowJo software (BD Biosciences).

### Sample collection and processing

Blood was collected from the mouse tail vein using Multivette 600 Serum gel tubes (Starstedt), rested at RT, and centrifuged at 10,000 rpm for 5 min following the provider’s instructions. Serum was recovered and stored at −20°C for neutralization assays and ELISAs. iLNs and spleens were harvested and placed in cold complete Dulbecco’s modified Eagle’s medium (DMEM, Corning, catalog no. T10014CV) containing 10% heat-inactivated FCS. All organs were kept on ice and immediately processed after collection. Organs were homogenized with a syringe plunger and filtered through a 40-μm cell strainer on ice. Cells from iLNs and spleens were resuspended in ice-cold complete RPMI and were used freshly for counting, culture, or staining.

### Fluorescent SARS-CoV-2 spike and RBD probe generation

A biotinylated soluble version of the SARS-CoV-2 spike protein consisting of the extracellular domain (amino acids V16 to K1211) and containing stabilizing proline substitutions at position K986P and V986P (Miltenyi Biotec, catalog no. 130-127-682) was used for flow cytometry experiments. Additionally, a biotinylated soluble version of the SARS-CoV-2 RBD spike protein (Miltenyi Biotec, catalog no. 130-127-458) was used for flow cytometry in Figures S2H and S2I. In separate tetramerization reactions the proteins were conjugated to streptavidin-PE (BioLegend, catalog no. 405203) and streptavidin-BV421 (BioLegend, catalog no. 405226), respectively, at a 1:4 molar ratio.^63^ Streptavidin-conjugated fluorophores were added sequentially in 4 steps, with 15-min incubation at 4°C between each step. Fluorescent spike or RBD protein tetramers were used freshly after conjugation.

### SARS-CoV-2 pseudovirus neutralization assays

SARS-CoV-2-nAbs were measured by diluting serum samples from naive (negative control) and immunized mice in MEM supplemented with 2% FCS, starting with a 1:10 dilution followed by 3-fold serial dilutions in 96-well plates. Monoclonal antibody (mAb) S309^64^ was included as a positive control. Each dilution of serum or mAb was incubated with an equal volume containing approximately 100 infectious units of replication-deficient, GFP-expressing vesicular stomatitis virus(rVSV-EGFP) pseudotyped with SARS-CoV-2 spike protein WA1/2020 strain (Wuhan/WIV04/2019, GISAID: EPI_ISL_402124), B.1.351 variant (Beta, GISAID: EPI_ISL_712096), or Omicron BA.5 variant (GISAID: EPI_ISL_12029894) for 1 h at 37°C. Subsequently, the mixture was incubated with Vero E6 cells (2 × 10^4^ cells/well) for 16 h and fixed with 2% PFA. The number of green spots was quantified using an Immunospot S6 device (C.T.L.). The 50% neutralization titer (NT50) was calculated as the half-maximal inhibitory concentration values using four-parameter nonlinear regression in GraphPad Prism.

### Generation of the IL-21-expressing CD40LB cell-based feeder cell line BaselGC-11

A murine interleukin-21 (IL-21) expressing CD40LB cell-based feeder cell line was generated following a strategy similar to the one described previously.^65^ In brief, parental CD40LB cells^66^ were maintained at 37°C/5% CO_2_ in DMEM (catalog no. D0819, Sigma-Aldrich) supplemented with 10% fetal bovine serum (FBS; catalog no. A5256701, Gibco), 1% MEM Non-Essential Amino Acids Solution (catalog no. 11140050, Gibco), 2 mg/mL G418 (catalog no. 10131027, Gibco), and 5 μg/mL puromycin (catalog no. A1113803, Gibco) until stable cell growth was achieved under these antibiotic selection conditions. Next, CD40LB cells were subjected to multiple rounds of transduction with a murine stem cell virus (MSCV)-based retroviral vector expressing murine IL-21 (pMSCV-mIL21-IRES2-mCD8α). Transduction was performed by spinoculation for 90 min at 32°C and 1,200 × *g* in the presence of 8 μg/mL Polybrene (catalog no. TR-1003-G, Sigma-Aldrich). Afterward, efficiently transduced cells were identified by staining for anti-mouse CD8a antibody (clone 53-6.7), and anti-mouse CD154 antibody (clone MR1) and mCD8α^+^ CD154^+^ double-positive single cells were fluorescence-activated cell sorting (FACS) sorted into 96-well plates using on an FACS Aria cell sorter (Becton Dickinson). Single feeder cells were expanded in DMEM supplemented with 10% FBS, 1% MEM Non-Essential Amino Acids, 0.5 mg/mL G418, and 2.5 μg/mL puromycin as described above. Individual wells were visually inspected to confirm growth of only one single colony prior to subsequent expansion.

A total of 30 expanded subclones were screened for their capacity to support the production of high amounts of IgG by individual GC B cells from mice infected with lymphocytic choriomeningitis virus 4 weeks prior to single GC B cell sorting. Out of the 30 feeder cell clones tested, we selected 1 clone, termed BaselGC-11, that consistently supported high antibody output from ∼20% of single cell-sorted GC B cells.

### Single GC B cell culture and assessment of IgG in culture supernatant

After thawing a frozen cell stock aliquot, BaselGC-11 cells were cultured and expanded for 10–14 days under the conditions described above while keeping them strictly subconfluent throughout. Next, puromycin and G418 supplementation was stopped and replaced by penicillin-streptomycin (100 U/mL, catalog no. 10378016, Gibco) for 72 h. Then, the cells were detached and irradiated with 10 Gy, and 5,000 DAPI-negative CD140a^+^ (clone APA5) cells (counted using a Cytek Aurora flow cytometer) were plated into 96-well U-bottom plates in 100 μL GC B cell medium (OptiMEM supplemented with 10% FBS Premium Plus [catalog no. A5669701, Gibco]), 1 mM sodium pyruvate (catalog no. 11360070, Gibco), 1% MEM Non-Essential Amino Acids Solution (catalog no. 11140050, Gibco), 0.5% MEM amino acids solution (catalog no. 11130051), 10 mM HEPES (catalog no. 15630080, Gibco), 2 mM l-glutamine (catalog no. 25030081, Gibco), 1% MEM vitamin solution (catalog no. 11120052, Gibco), 1% insulin-transferrin-selenium-ethanolamine (catalog no. 51500056, Gibco), 1% HT supplement (catalog no. 11067030, Gibco), 2 g/L d-glucose (catalog no. A2494001, Gibco), 1× Lonza MycoZap Plus-PR (catalog no. VZA-2021, Lonza), 0.1% antioxidant supplement (catalog no. A1345, Sigma-Aldrich), and 50 μM 2-mercaptoethanol (catalog no. 31350010, Gibco) and cultured for 18–24 h at 37°C/5% CO_2_. Then, IL-4 (catalog no. 214-14-20UG, Peprotech) and heat-inactivated lipopolysaccharides (catalog no. L6511, Sigma-Aldrich) were added in 150 μL GC B cell medium to a final concentration of 4 ng/mL and 30 μg/mL, respectively, as previously described.^67^ Single GC B cells were sorted onto the feeder cell layer in individual 96-well plates using an FACS Aria cell sorter (Becton Dickinson). Culture supernatants were collected after 10 days of culture at 37°C in a 5% CO_2_ atmosphere and were screened for IgG content as described below. The number of wells with a detectable concentration of >10 ng/mL IgG was 145/504 (28.7%) for GC B cells collected from iLNs on day 14 and 139/504 for the GC B cells collected from iLNs on day 42 (27.6%).

### ELISA-based quantification of IgG in the supernatant of single GC B cell cultures

In the single GC B cell cloning experiment, the culture wells containing ≥10 ng/mL IgG or more were identified by ELISA. In brief, 96-well high-binding plates (Greiner) were coated overnight at 4°C with 0.5 μg/mL goat anti-mouse IgG (H + L) (Jackson ImmunoResearch) diluted in coating buffer (15 mM Na_2_CO_3_, 35 mM NaHCO_3_, pH 9.6). The following day the plates were blocked with blocking buffer (PBS/0.05% Tween/5% milk; also used as buffer in the subsequent steps) for 1.5 h at RT. Subsequently, feeder culture supernatants were added (pre-diluted 20-fold in blocking buffer). To generate a standard curve for quantification, purified KL25 mAb (supplied by the European Virus Archive) was 3-fold serially diluted in duplicates starting from 10 μg/mL. Duplicate blank feeder culture supernatants that were kept without the addition of GC B cells were used to determine technical backgrounds. After overnight incubation at 4°C, the plates were washed three times with PBS-0.05% Tween 20 (PBS-T). Bound antibody was detected by incubating the plates with HRP-conjugated goat anti-mouse IgG (H + L) (Jackson Immunoresearch). After 45 min of incubation at RT, the binding signal was developed using 2,2′-azino-bis(3-ethylbenzothiazoline-6-sulfonic acid) (ABTS; Sigma-Aldrich), and the reaction was stopped using 1% SDS dissolved in water. Optical density at 405 nm values were measured using a TECAN Infinite M Plex plate reader. Antibody concentrations from supernatants were calculated in GraphPad Prism (version 10.2.2) by fitting the values to a sigmoidal 4PL standard curve interpolated with the serial dilution measurements obtained from the reference standard. Supernatants exceeding the upper range of quantification were further diluted and reanalyzed to obtain accurate IgG concentrations. A total of 103 single-cell culture supernatants contained ≥3 μg/mL IgG and were further screened for SARS-CoV-2 Wuhan-Hu-1 spike S1 domain binding by ELISA as described below. Thirty-seven supernatants, 17 from day 14 clones and 20 from day 42 clones, exhibited detectable binding, and when assessed for Wuhan-Hu-1-neutralizing activity, 15 clones from each time point tested positive and were further studied as reported in Figure 5E.

### Spike protein and adenovirus vector ELISAs

High-binding 96-well flat bottom plates (Sarstedt AG & Co. KG) were coated with 50 ng recombinant SARS-CoV-2 spike S1 subunit protein per well in 50 μL coating buffer overnight at 4°C. Plates were washed twice with PBS-T, then blocked with 200 μL 5% BSA/PBS-T at RT for 45 min. Twofold serial dilutions of serum samples in blocking solution were performed after washing five times with PBS-T. Plates were incubated at 37°C for 1 h and washed five times with PBS-T. Peroxidase-conjugated polyclonal anti-mouse antibody (1:2,000 in blocking solution; Jackson, catalog no. 115-035-062) was added, and the plates were incubated at 37°C for 60 min. After washing five times with PBS-T, horseradish peroxidase (HRP) activity was detected using ABTS as a chromogen (Pierce), and the absorbance was measured at 405 nm using the Saphirell plate reader (Tecan). Arbitrary units were computed as ln(1,000 × A491 nm); the limit of detection was defined as the maximum value reached by negative controls.

For determination of anti-adenovirus vector antibody titers, high-binding 96-well flat bottom plates (Sarstedt AG & Co. KG) were coated with 1 × 10^8^ vp/well of heat-inactivated ChAdOx1 (65°C, 10 min) and incubated overnight at 4°C in coating buffer. Plates were washed twice with PBS-T and subsequently blocked with 5% BSA/PBS-T for 45 min at RT. After washing five times with PBS-T, mice sera were diluted 2-fold in blocking solution, then incubated for 1 h at 37°C. Plates were washed five times with PBS-T and incubated with peroxidase-conjugated anti-mouse antibody (1:2,000 in blocking solution, Merck KGaA, catalog no. A9044). Plates were incubated at 37°C for 70 min and subsequently washed five times with PBS-T. Colorimetric reaction was started by the addition of 100 μL of an σ-phenylenediamine-dihydrochloride substrate solution (1 tablet in 0.05 M phosphate-citrate buffer; Merck KGaA, catalog no. P3804). The reaction was stopped with 1 M sulfuric acid, and absorption at a wavelength of 491 nm was measured using the SPECTROstar Nano (BMG LABTECH GmbH).

### Immunohistochemistry

Mouse LNs were infused overnight in 4% PFA, embedded in paraffin, and 2-μm sections were mounted on a glass slide. For fluorescent staining of GL7, GFP, and B220, tissue sections were incubated with anti-mouse IgG Fab fragments (Jackson ImmunoResearch, catalog no. 115-007-003) and PBS/2.5% normal goat serum (Jackson ImmunoResearch, catalog no. 005-000-4121) to avoid non-specific binding. Sections were then incubated overnight with a rat anti-GL7 antibody (eBioscience, catalog no. 14-5902-81) and a rabbit anti-GFP antibody (Cell Signaling Technology, catalog no. 2956S), and specific binding was visualized using appropriate species-specific Al488- and Al555-conjugated secondary antibodies, respectively. For co-staining with B220, sections were incubated with 10% normal rat serum (Jackson ImmunoResearch, catalog no. 012-000-001) to avoid non-specific binding, followed by 1-h incubation with a rat anti-B220 Al647 conjugate (eBioscience, catalog no. 102707 and labeling kit, Life Technologies, catalog no. A20186). Nuclear counterstaining was performed with DAPI (Life Technologies, catalog no. D3571). Stained sections were scanned using the Panoramic 250 FLASH II (3DHISTECH) whole-slide scanner at a 0.221-μm/pixel resolution.

### Limitations of the study

We acknowledge that the AID^rep^ fate-mapping approach labels only about 30% of GC B cells.^68^ Accordingly, this experimental approach underestimates absolute cell counts, but relative differences between groups should be unaffected by this technical shortcoming. An additional limitation of our study consists in the prime-boost interval tested. In the context of clinical use, homologous ChAdOx1 and heterologous mRNA/ChAdOx1 vaccinations were mostly administered at 3-month intervals,^21^^,^^22^^,^^23^^,^^24^^,^^25^^,^^26^^,^^27^^,^^34^ while mRNA homologous boost was generally conducted after 1 month.^69^ For the purpose of head-to-head comparisons in our study, a uniform timing was used, but we are aware that the interval between prime and boost has the potential to profoundly impact the dynamics of GC responses.^34^^,^^70^ Furthermore, we have compared and discussed mRNA-1273 and ChAdOx1 as representatives of RNA- and adenoviral vector-based vaccines that have been used during the COVID-19 pandemic. We acknowledge that the two vaccines differ also in antigenic properties of the encoded SARS-CoV-2 spike protein. Unlike mRNA-1273, the ChAdOx1 vaccine construct lacks prefusion-stabilizing mutations.^56^ Of note, however, the expression of the unmodified spike sequence from the ChAdOx1 vector results in the cell surface expression of spike protein that adopts predominantly a trimeric prefusion conformation.^71^^,^^72^ Accordingly, the introduction of prefusion-stabilizing mutations into the ChAdOx1-vectored spike protein was reported not to augment spike-specific antibody responses.^73^ Last but not least, the dosage of vaccines can substantially influence immune responses, and the accurate extrapolation of human vaccine dosing to small animal models is challenging.

## Data availability

Raw data of the experimental results reported in this study have been deposited with Zenodo and are publicly available as of the date of publication under the https://doi.org/10.5281/zenodo.14676975.

## Acknowledgments

We wish to thank Karsten Stauffer for excellent animal handling and care and Min Lu and Karen Cornille for technical support. We thank Florian Banderet, Sabine Egli, and Bertha Menigoz from the Medical Polyclinic of the Basel University Hospital for providing mRNA-1273 vaccine leftovers; Onur Boyman and Yves Zurbuchen for providing protocols for the detection of spike-specific B cells; Jürg Schwaller and Rathick Sivalingam for their help and generous sharing of reagents for MSCV production; Gabriel D. Victora and Alvaro Hobbs for advice on single GC B cell cultures; the DBM flow cytometry core facility for FACS sorting; Robert Ivanek from the DBM bioinformatics core facility for advice on statistics; Gert Zimmer for providing GFP-expressing VSV vectors for pseudovirus neutralization assays; Cynthia Saadi for technical assistance with immunohistochemistry; and the entire Experimental Virology group for helpful discussions. We also gratefully acknowledge support from the 10.13039/501100000739University of Southampton Coronavirus Response Fund (M. Crispin). This work was supported by the 10.13039/501100001711Swiss National Science Foundation (grant nos. 4078P0_198431/1 and 310030_215043/1 to D.D.P.), the European Union’s Horizon 2020 research and innovation program under Marie Skłodowska-Curie grant agreement (no. 812915 to D.D.P.), and the EU-H2020-MSCA-COFUND EURIdoc programme (grant no. 101034170 to D.D.P).

## Author contributions

M. Ciancaglini, J.F., C.S., M.D., D.F., D.W., A.L.K., F.J., I.W., I.V., A.P., M.L.N., M. Crispin, D.M., F.K., and D.D.P. designed the experiments. M.Ci., J.F., C.S., M.D., D.F., D.W., A.L.K., F.J., I.W., I.V. and M.L.N. conducted the experiments and acquired and analyzed the data. M. Ciancaglini, J.F., C.S., F.K., and D.D.P. wrote the manuscript.

## Declaration of interests

D.D.P. is a founder of, consultant to, and shareholder in Hookipa Pharma Inc., commercializing arenavirus-based vector technology, and he as well as D.M. are listed as inventors on corresponding patents.

## Supplemental information

Document S1. Figures S1–S4

Document S2. Article plus supplemental information
