# Supplementary material for: mRNA prime-boost vaccination promotes clonal continuity in germinal center reactions and broadens SARS-CoV-2 variant coverage
Source: Mol Ther. 2025 Aug 8;33(11):5453–69. doi: 10.1016/j.ymthe.2025.08.008 (PMC12628176; doi:10.1016/j.ymthe.2025.08.008)
Supplement: Document S1. Figures S1–S4 [file mmc1.pdf]

YMTHE, Volume *33*

Supplemental Information

mRNA prime-boost vaccination promotes clonal continuity in germinal center reactions and broadens SARS-CoV-2 variant coverage

Matias Ciancaglini, Jonas Fixemer, Cemre Seven, Mirela Dimitrova, Davide Finozzi, Denice Weklak, Anna Lena Kastner, Franziska Jönsson, Ingrid Wagner, Ilena Vincenti, Anneli Peters, Maddy L. Newby, Max Crispin, Doron Merkler, Florian Kreppel, and Daniel D. Pinschewer

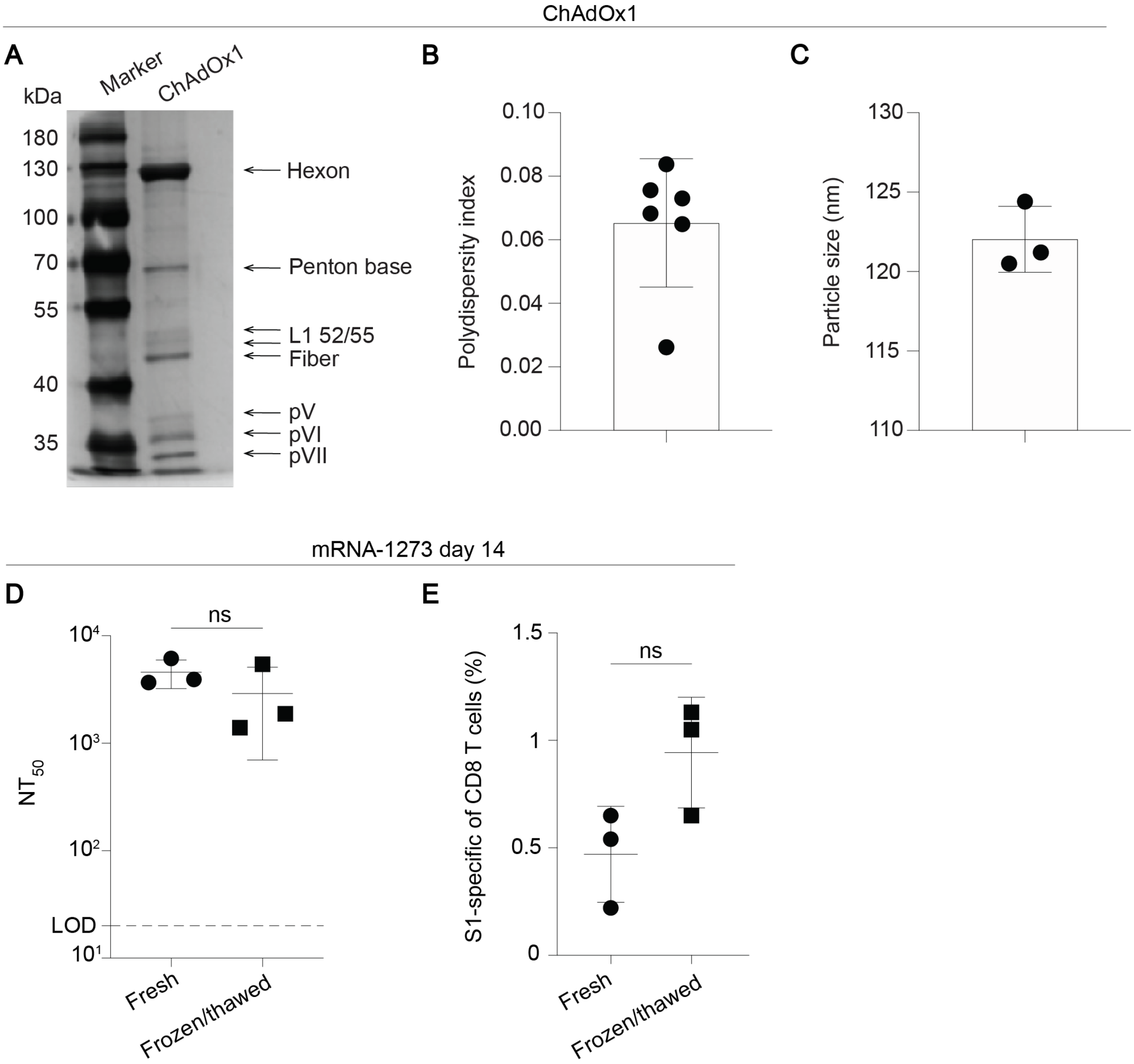

Figure S1. Determination of the purity and homogeneity of ChAdOx1 vector preparations and impact of freeze-thawing on mRNA-1273 immunogenicity. (A) A total of 1×10^{10} viral particles (VPs) were denatured at 80 °C for 5 min in SDS sample buffer (250 mM Tris-HCl, pH 6.8, 2% SDS, 6% glycerol, 50 mM DTT, 0.01% bromophenol blue) and separated by SDS-PAGE on an 8% polyacrylamide gel. Gels were subsequently silver-stained according to Blum *et al.*[67]. The left lane of the gel contains the molecular size marker as indicated, the right lane contains the vector sample. The vector proteins are indicated by black arrows. They were identified based on their molecular weight as determined by mass spectrometry[68]. (B) The polydispersity index (PDI) remained well below 0.2, the value commonly used as a cut-off for particle aggregation. (C) Dynamic light scattering (DLS) measurements performed with 5×10^{10} VPs revealed a consistent hydrodynamic diameter of ~122 nm, characteristic of adenovirus particles. (D,E) We immunized mice with mRNA-1273 that was either used freshly as supplied by the

provider or frozen to -80°C and thawed again prior to administration (as performed for the purpose of aliquotting, storage and subsequent use in all other experiments of this report). Immune responses were analyzed 14 days after immunization. Serum neutralizing antibody titers against SARS-CoV-2 Wuhan-Hu-1 (D) and frequencies of spike-specific CD8 T cells in blood (E) are reported. Two-tailed unpaired Student's *t* tests were performed for statistical analysis in (D) and (E). $p > 0.05$ was considered not statistically significant (ns).

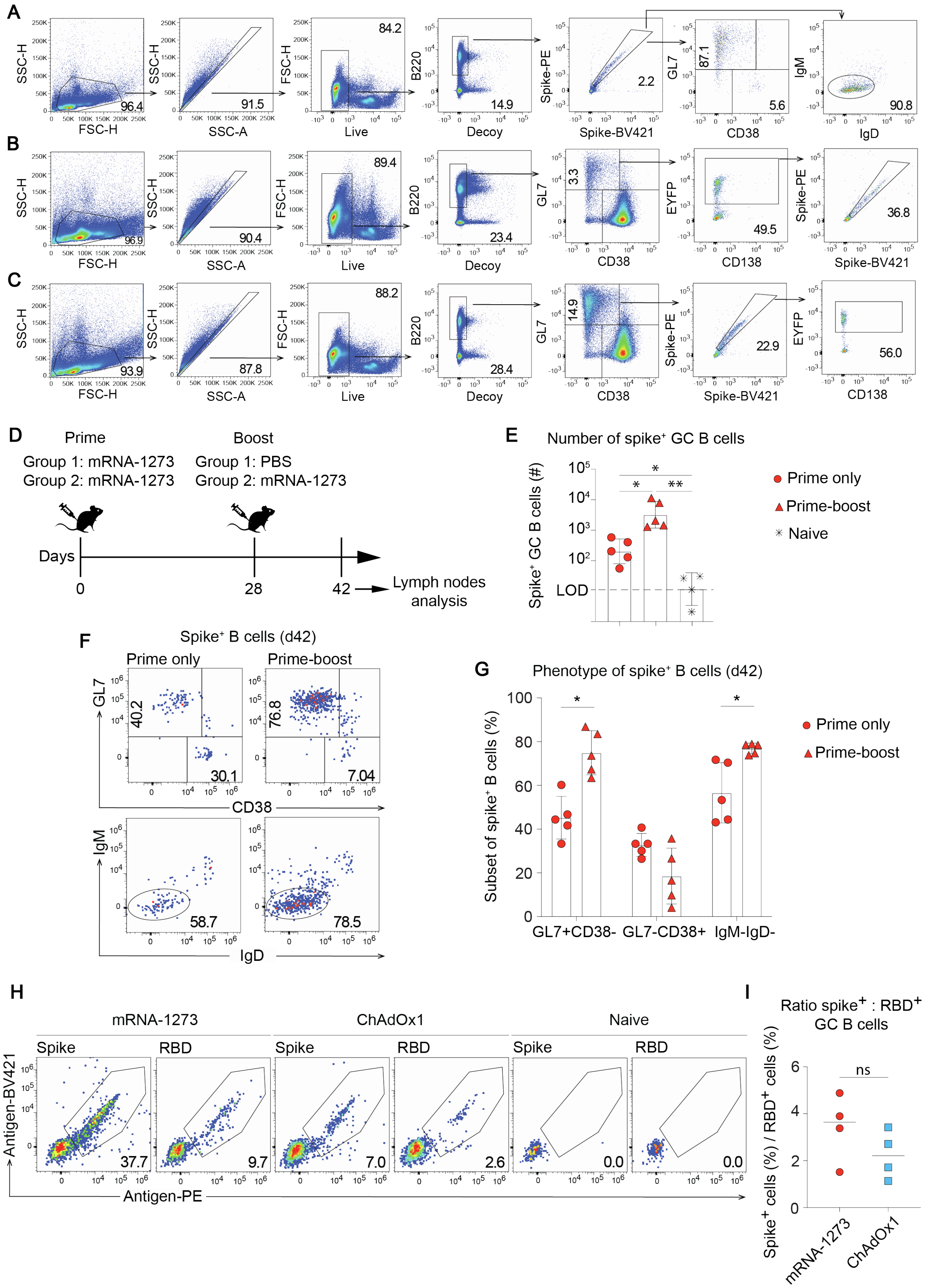

Figure S2. Flow cytometry gating strategies, longevity of mRNA-1273-induced germinal center responses and detection of spike-specific GC B cells using different fluorescent probes. (A)

Gating strategy for the analysis of spike$^+$ B cells, their phenotype, frequency and number by means of fluorescently labelled recombinant spike tetramers and antibodies against cellular surface markers. Lymphocytes in iLN single cell suspensions were identified based on FSC-A and SSC-A. Single cells were selected based on SSC-A and SSC-H. Live cells were identified based on negative staining with Zombie NIR viability dye. Total B cells were then identified from live cells as B220$^+$. A decoy probe (fluorophore-conjugated streptavidin) was used to exclude cells that nonspecifically bound to streptavidin. The antigen specificity of B cells was determined based on their binding to fluorescently labeled spike tetramers. B cell subsets were identified based on expression of GL7, CD38, IgM and IgD. (B) Gating strategy for the analysis of spike$^+$ B cells within the EYFP$^+$ GC B cell compartment. (C) Gating strategy for the analysis of fate-mapped (EYFP$^+$) B cells within the spike$^+$ GC B cell compartment. (D) Mice were primed with mRNA-1273 on d0 and boosted with PBS or mRNA-1273 on d28. iLNs were collected on d42 for analysis by flow cytometry. (E) Total number of spike$^+$ GC B cells in iLNs on d42. (F) Exemplary FACS plots of GC B cells (GL7$^+$ CD38$^-$, top row) and class-switched B cells (IgD$^-$ IgM$^-$, bottom row) on d42. (G) Percentages of GC phenotype (GL7$^+$ CD38$^-$), memory phenotype (GL7$^-$ CD38$^+$) and class-switched (IgM$^-$ IgD$^-$) spike$^+$ B cells. (H,I) We immunized mice with mRNA-1273 or ChAdOx-1 on d0 and on d14 we analyzed GC B cells in draining lymph nodes. (H) Exemplary FACS plots detecting spike-binding and RBD-binding GC B cells in the two groups. (I) Ratio of spike$^+$ to RBD$^+$ GC B cells of individual mice. One-way ANOVA with Tukey's post-test was performed in (E), unpaired two-tailed Student's t-test were used in (G) and (I). $p < 0.05$: *; $p < 0.01$: **; $p > 0.05$ was considered not statistically significant.

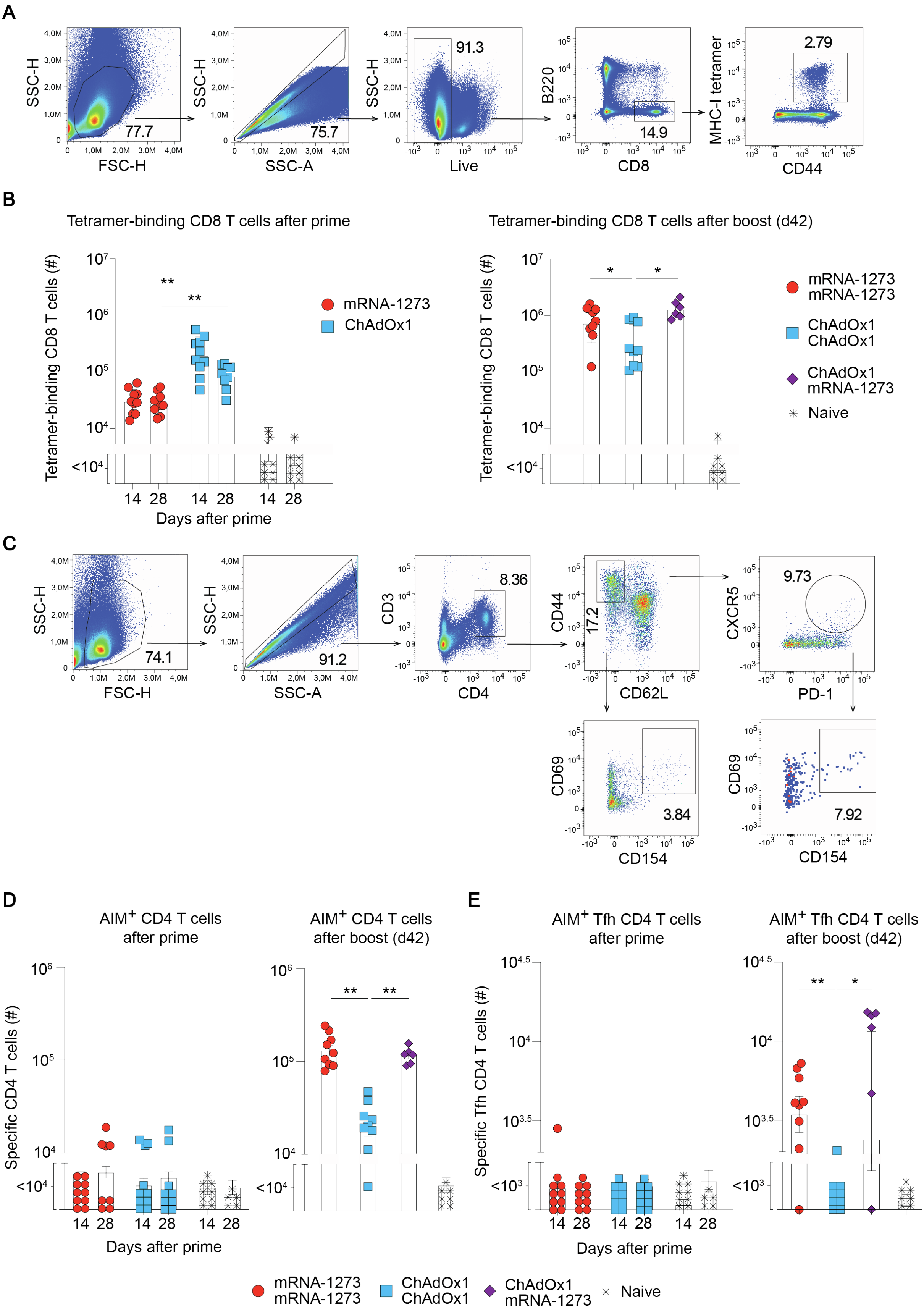

Figure S3. Spike-specific CD8 and CD4 T cell responses elicited by the different vaccination regimens. (A) Gating strategy for the analysis of spike-specific CD8 T cells in spleen by means of MHC-

I tetramers and surface markers. (B) Total number of spike-specific CD8 T cells in spleens of animals vaccinated with different regimens at the indicated time points after vaccination. (C) Gating strategy for the identification of spike-specific CD4 T cells by means of the AIM assay. (D,E) Total number of spike-specific CD4 T cells (D) and of spike-specific Tfh CD4 T cells (E) in the spleen of mice immunized with different regimens at the indicated time points. For statistical analysis one-way ANOVA with Tukey's post-test was performed. $p < 0.05$: *; $p < 0.01$: **; $p > 0.05$ was considered not statistically significant and is not indicated.

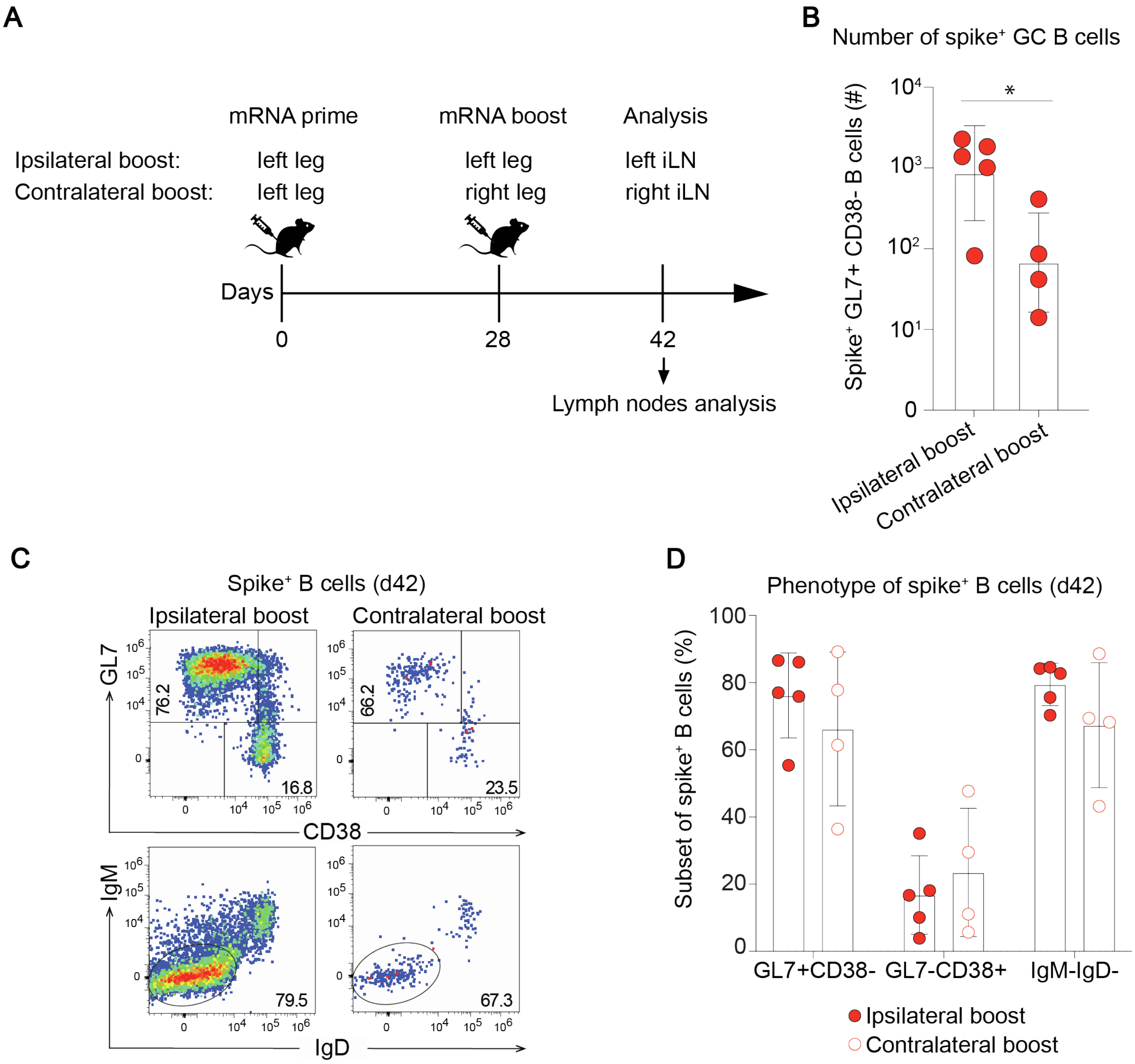

Figure S4. Comparison of spike+ B cell responses to ipsilateral and contralateral mRNA-1273 prime-boost. (A) Experimental design. Mice were primed on d0 with mRNA-1273 into the left leg and boosted on d28 with mRNA-1273 into either the left leg (ipsilateral boost) or into the right leg (contralateral boost). The iLN draining the site of the boost was collected on d42 for analysis by flow cytometry. (B) Total numbers of spike+ GC B cells in the respective inguinal lymph nodes on d42. (C) Concatenated FACS plots of spike+ B cells. (D) Percentages of GC phenotype (GL7+ CD38-), memory phenotype (GL7- CD38+) and class-switched (IgM- IgD-) spike+ B cells. For statistical analysis unpaired two-tailed Student's t-test wewe performed in (B) and (D). $p < 0.05$: *; $p > 0.05$ was considered not statistically significant and is not indicated.
